# Deep Learning Algorithms for Screening and Diagnosis of Systemic Diseases Based on Ophthalmic Manifestations: A Systematic Review

**DOI:** 10.3390/diagnostics13050900

**Published:** 2023-02-27

**Authors:** Wai Cheng Iao, Weixing Zhang, Xun Wang, Yuxuan Wu, Duoru Lin, Haotian Lin

**Affiliations:** 1 State Key Laboratory of Ophthalmology, Zhongshan Ophthalmic Center, Sun Yat-sen University, Guangdong Provincial Key Laboratory of Ophthalmology and Vision Science, Guangdong Provincial Clinical Research Center for Ocular Diseases, Guangzhou 510060, China; 2 Hainan Eye Hospital and Key Laboratory of Ophthalmology, Zhongshan Ophthalmic Center, Sun Yat-sen University, Haikou 570311, China; 3 Center for Precision Medicine and Department of Genetics and Biomedical Informatics, Zhongshan School of Medicine, Sun Yat-sen University, Guangzhou 510060, China

**Keywords:** systemic diseases, screening tests, ocular manifestations, deep learning, artificial intelligence

## Abstract

Deep learning (DL) is the new high-profile technology in medical artificial intelligence (AI) for building screening and diagnosing algorithms for various diseases. The eye provides a window for observing neurovascular pathophysiological changes. Previous studies have proposed that ocular manifestations indicate systemic conditions, revealing a new route in disease screening and management. There have been multiple DL models developed for identifying systemic diseases based on ocular data. However, the methods and results varied immensely across studies. This systematic review aims to summarize the existing studies and provide an overview of the present and future aspects of DL-based algorithms for screening systemic diseases based on ophthalmic examinations. We performed a thorough search in PubMed^®^, Embase, and Web of Science for English-language articles published until August 2022. Among the 2873 articles collected, 62 were included for analysis and quality assessment. The selected studies mainly utilized eye appearance, retinal data, and eye movements as model input and covered a wide range of systemic diseases such as cardiovascular diseases, neurodegenerative diseases, and systemic health features. Despite the decent performance reported, most models lack disease specificity and public generalizability for real-world application. This review concludes the pros and cons and discusses the prospect of implementing AI based on ocular data in real-world clinical scenarios.

## 1. Introduction

Deep learning (DL) is a state-of-the-art subset of machine learning that allows computers to automatically learn the features from raw data. It comprises multiple processing layers that transform the data into stratified abstract levels to achieve specific tasks [1]. In recent years, DL has significantly advanced in various fields, such as visual recognition and natural language processing. With its ability to unveil intrinsic characteristics from high-dimensional data, DL has also been widely applied in medical AI to develop disease screening and diagnosis algorithms.

Ophthalmology is a pioneer in the field of medical artificial intelligence (AI). The eye is informative and accessible due to its inherent anatomical features. As an organ located on the body surface, most examinations can be done non-intrusively. Furthermore, the complex anatomy comprising various cells and tissues allows the generation of multimodal data through diverse exam methods. These features provide data scientists and ophthalmologists with easily collectible data of good quality and quantity, making the eye a prime candidate for medical AI.

With the FDA’s approval of IDx-DR [2] as the first autonomous AI system for marketing, the development of ophthalmic AI preemptively entered a new era. Various DL algorithms have been developed for ophthalmic purposes [3], such as for diagnosing diabetic retinopathy [4], glaucoma [5], and age-related macular degeneration [6] based on color fundus photographs. Other forms of ocular examinations were also studied, such as color-coded corneal maps for detecting keratoconus [7] and corneal confocal microscopy for diabetic corneal neuropathy [8]. Beyond ocular diseases, ophthalmic manifestations can also indicate underlying systemic conditions. The innate anatomical structure of the eye provides a window for observing neural and vascular systems in vivo, making it possible to intuitively record physiological and pathological changes in the body. As a result, systemic diseases that induce vascular and neural impairment could present as ophthalmic complications, such as diabetic retinopathy and anemic retinopathy. Recent studies have proven that non-specific pathologies, namely vessel narrowing and retinal thinning, were predictive of systemic disease status and long-term prognostics [9,10]. Aside from the static presentations, there was also evidence for diagnosing neurodegenerative diseases based on specific eye movements [11]. With the help of the ever-developing DL technology, these characteristics can now be identified and utilized for screening and diagnosing purposes.

DL algorithms based on ocular manifestations have opened up new opportunities for managing systemic diseases in rapid, non-invasive manners. Most common ailments have been studied for their correlation with the eye, resulting in a growing number of diverse DL algorithms. However, these AI models are still immature in various aspects to be applied in clinical scenarios. This review aims to conclude and summarize the current DL-based AI models for detecting systemic diseases based on ocular manifestations. We intend to provide a well-rounded overview of the previous works as a reference for future studies.

## 2. Materials and Methods

This systemic review is conducted and reported in accordance with the Preferred Reporting Items for Systematic Reviews and Meta-Analysis (PRISMA) 2020 guidelines. We searched PubMed^®^, Embase, and Web of Science for English-language articles published until August 2022. The keywords were designed based on three elements: (1) ocular characteristics, (2) systemic diseases, and (3) artificial intelligence methods. The detailed search strategies are listed in Supplementary Table S1.

### 2.1. Selection Criteria

Studies were deemed eligible if they (1) were retrospective, cross-sectional, or prospective studies; (2) applied deep learning algorithms to analyze ocular characteristics for identifying systemic diseases; (3) reported the performance of the algorithms with metrics such as accuracy, area under the receiver operating characteristic curve (AUC), sensitivity, and specificity for binary outcomes or mean absolute error (MAE) and R square for regression models; (4) included validation experiment. Reviews, case reports, letters, comments, editorials, meta-analyses, and animal studies were excluded from this systematic review. Full-text revision and reference screening were performed on all qualified articles by two authors (W.C.I. and W.Z.).

### 2.2. Data Extraction and Quality Assessment

Pertinent data were extracted according to a pre-designed table by W.Z. and W.C.I. Information including the author, publication year, ocular data, DL model, training dataset, testing/validation dataset, external validation, systemic health features/diseases, outcome, and model performance were collected from the selected studies. The Quality Assessment of Diagnostic Accuracy Studies 2 (QUADAS-2) [12] was applied to evaluate the quality and the risk of bias of the included studies.

## 3. Results

### 3.1. Study Selection

**Figure 1:**
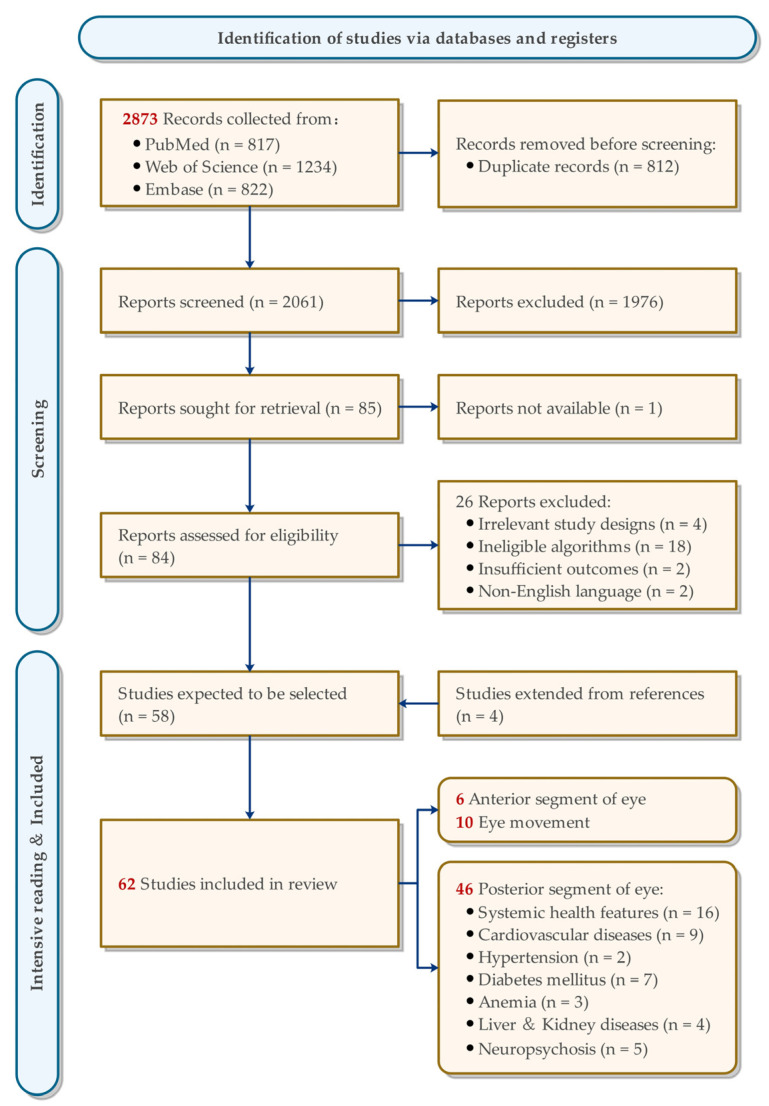
Flowchart of the study selection process.

The flowchart of the selection process is demonstrated in Figure 1. In the searching stage, 2873 articles were identified from the three target databases, with 817 from PubMed, 822 from Embase, and 1234 from Web of Science. After removing 812 duplicates, 1976 reports were excluded by screening the title and abstract. In the 85 studies entering the full-text screening stage, one was unavailable and 26 others were eliminated for various reasons. With the addition of the four studies extended from references, 62 studies were included in this systematic review. The included articles are summarized in Table 1, Table 2 and Table 3.

**Figure 2:**
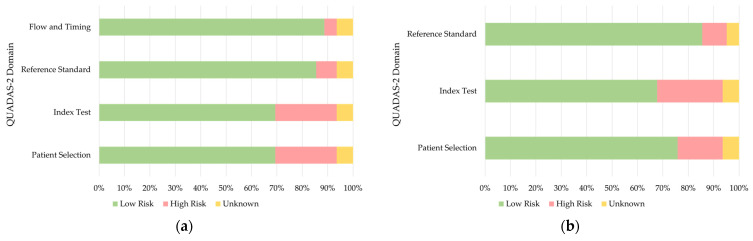
Stacked bar chart of the QUADAS-2 analysis: (**a**) Risk of Bias; (**b**) Applicability Concerns.

The results of the risk of bias evaluation using QUADAS-2 is demonstrated in Figure 2. Most of the studies included had low risk in all categories, while larger proportions of high risk were found in patient selection and index test. The detailed results are shown in Supplementary Table S2.

**Table 1:** Summary of deep learning algorithms identifying systemic diseases from anterior segment of the eye.

Author, Year	Ocular Data	DL Model	Training Dataset	Testing/Validation Dataset	External Validation	Systemic Health Features/Diseases	Outcome	Performance ^1^
Babenko et al., 2022 [13]	External eye images	DLS	EyePACS (CA): 126,066 patients, 290,642 images	19,766 patients, 41,928 images	Set A: EyePACS (non-CA): 27,415 patients, 53,861 images; Set B: EyePACS (non-CA): 5058 patients, 9853 images; Set C: 10,402 patients, 19,763 images; Set D: 6266 patients, 12,751 images	HbA1c Total cholesterol Triglycerides	Binary Binary Binary	AUC: 73.4% AUC: 62.3% AUC: 67.1%
Li et al., 2022 [14]	Conjunctival images	HMT-Net	68 patients, 405 images; 62 HC, 206 images	5-fold cross-validation	N/A	T2DM	Binary	Sensitivity: 78.70% Specificity: 69.08% Accuracy: 75.15% AUC: 0.82
Preston et al., 2022 [15]	CCM images	ResNet-50	65 HC images, 63 T1DM, 89 T2DM, 28 prediabetes	Test: 15 HC, 11 T1DM, 10 T2DM, 4 prediabetes; Validation: 10 HC, 14 T1DM, 42 T2DM, 18 prediabetes	N/A	DPN	Ternary	1. Healthy: *F*_1_-score: 0.91 2. No DPN: *F*_1_-score: 0.88 3. DPN: *F*_1_-score: 0.91
Scarpa et al., 2020 [16]	CCM images	CNN	40 patients, 240 images; 40 HC, 240 images	10 patients, 60 images; 10 HC, 60 images; 5-fold cross-validation	N/A	DPN	Binary	Sensitivity: 98% Specificity: 94% Accuracy: 96%
Althnian et al., 2021 [17]	Scleral images	VGG-16	24 images of patients, 44 images of HC	N/M	N/A	Neonatal jaundice	Binary	Accuracy: 79.03% *F*_1_-score: 70.73% AUC: 69.67%
Lv et al., 2021 [18]	Scleral images	U-Net, Resnet-18, MIL model	576 participants, 4608 images	145 participants, 1160 images; 5-fold cross-validation	N/A	PCOS	Binary	AUC: 0.979 Accuracy: 0.929 *F*_1_-score: 0.934

^1^ Only the best performance are presented when there was more than one model. Metadata-based models and hybrid models are not presented in this table. EyePACS, eye picture archive communication system; HbA1c, glycated hemoglobin; AUC, area under curve; HC, healthy control; N/A, not applicable; T2DM, type 2 diabetes mellitus; CCM, corneal confocal microscopy; T1DM, type 1 diabetes mellitus; DPN, diabetic peripheral neuropathy; CNN, convolutional neural network; N/M, not mentioned; PCOS, polycystic ovary syndrome.

### 3.2. Algorithms Based on the Anterior Segment of the Eye

Most abnormalities of the external eye can be observed intuitively. These manifestations could provide easy access to several systemic diseases and were proven accessible with deep learning models. Babenko et al. [13] developed the DL algorithms based on external eye images taken with fundus cameras to distinguish hemoglobin A1c (HbA1c) ≥ 9% and lipid levels. The former achieved the highest AUC of 0.67 to 0.73, though the latter lacked significance. The models mainly focused on the nasal and temporal scleral areas, indicating that the clue for diagnosis may be conjunctival vessels. The work of Li et al. [14] proved that diabetes could be identified from conjunctival images with an accuracy of 75.15%.

Jaundice is also a distinct symptom often observed from the external eye. Using slit lamp photos as input, Xiao et al. [19] achieved AUCs over 0.90 in diagnosing liver cirrhosis and liver cancer. Another study focusing on neonatal jaundice [17] also attained an accuracy of 79.03% based on smartphone-captured images. On the other hand, the model built by Lv et al. [18] detected polycystic ovary syndrome (PCOS) based on sectioned scleral images. The model attained an accuracy and AUC over 0.90 by focusing on thick and foggy blood vessels in the sclera, which could be caused by sex steroid changes in the patients.

The cornea is densely innervated by the ophthalmic branch of the trigeminal nerves. Corneal confocal microscopy (CCM) allows for non-invasive quantification of the small corneal nerve fibers, providing a rapid evaluation method for various diseases. With CCM images as input, two DL algorithms were developed for the early diagnosis of diabetic neuropathy, one achieving an accuracy of 96% [16] and the other having an *F*_1_-score of 0.91 [15]. The Grad-CAM highlighted the absence of nerve fibers in the CCM images, showing that the models are explainable despite the relatively small datasets.

### 3.3. Algorithms Based on the Posterior Segment of the Eye

The retina provides a window for directly observing neurovascular structures in vivo based on its natural anatomical features. The development of retinal imaging technologies such as color fundus photographs and ultra-widefield fundus (UWF) imaging enabled intuitive en-face records of retinal pathologies. Additionally, optical coherence tomography (OCT) with the interferometry technique allows cross-sectional imaging of the multiple layers of the retina. The multimodal retinal data generated from various imaging methods creates an ideal platform for building DL algorithms for diagnosing systemic diseases.

**Table 2:** Summary of deep learning algorithms identifying systemic diseases from posterior segment of the eye.

Author, Year	Ocular Data	DL Model	Training Dataset	Testing/Validation Dataset	External Validation	Systemic Health Features/Diseases	Outcome	Performance ^1^
Betzler et al., 2021 [20]	Retinal images	VGG-16	SEED: 7969 participants, 137,511 images	1987 participants, 34,659 images	N/A	Gender	Binary	AUC: 0.94
Corbin et al., 2022 [21]	Fundus images	EfficientNet	14,711 participants for all datasets; 18,000 images for training	Validation: 3860 images; Test: 3877 images	N/A	Age SBP DBP BMI Sex (image) Sex (individual) APOE4 (image) APOE4 (individual)	Regression Regression Regression Regression Binary Binary Binary Binary	*R*^2^: 0.778, MAE: 3.24 *R*^2^: 0.229, MAE: 10.94 *R*^2^: 0.227, MAE: 6.80 *R*^2^: 0.032, MAE: 3.99 AUC: 0.84 AUC: 0.85 AUC: 0.47 AUC: 0.50
Gerrits et al., 2020 [22]	Retinal images	MobileNet-V2	Qatar Biobank: 1800 participants, 7200 images	Validation: 600 participants, 2400 images Test: 600 participants, 2400 images	N/A	Age Sex SBP DBP HbA1c BMI RFM Glucose Insulin SHBG Estradiol Testosterone Tch HDL LDL Tg Smoking status	Regression Binary Regression Regression Regression Regression Regression Regression Regression Regression Regression Regression Regression Regression Regression Regression Binary	MAE: 2.78, *R*^2^: 0.89 AUC: 0.97 MAE: 8.96, *R*^2^: 0.40 MAE: 6.84, *R*^2^: 0.24 MAE: 0.61, *R*^2^: 0.34 MAE: 4.31, *R*^2^: 0.13 MAE: 5.68, *R*^2^: 0.43 MAE: 1.06, *R*^2^: 0.12 MAE: 7.15, *R*^2^: −0.04 MAE: 21.09, *R*^2^: 0.06 MAE: 154.18, *R*^2^: −0.03 MAE: 3.76, *R*^2^: 0.54 MAE: 0.75, *R*^2^: 0.03 MAE: 0.31, *R*^2^: 0.05 MAE: 0.72, *R*^2^: −0.03 MAE: 0.49, *R*^2^: 0.03 AUC: 0.78
Hu et al., 2022 [23]	Retinal images	DL	UK Biobank: 11,052 participants, 19,200 images	35,834 participants	N/A	Retinal age	Regression	MAE: 3.55
Khan et al., 2022 [24]	Retinal images	DenseNet-201	760 participants for all datasets; 1021 images for training	256 images for testing	N/A	Gender ARB Smoking status ACEi LDL Hypertension HDL Cardiac disease HbA1c Age Aspirin Ethnicity	Binary Binary Binary Binary Binary Binary Binary Binary Binary Binary Binary Binary	AUC: 0.852 AUC: 0.783 AUC: 0.732 AUC: 0.815 AUC: 0.766 AUC: 0.687 AUC: 0.756 AUC: 0.7 AUC: 0.708 AUC: 0.902 AUC: 0.696 AUC: 0.926
Kim et al., 2020 [25]	Retinal images	ResNet-152	155,449 participants for all datasets; 216,866 HC images for training	Validation: 2436 HC images; Test: 24,366 HC images, 40,659 hypertension, 14,189 DM, 113,510 smoking	N/A	Age Sex	Regression Binary	MAE: 3.06, *R*^2^: 0.92 AUC: 0.969
Korot et al., 2021 [26]	Retinal images	CFDL	UK Biobank: 84,743 patients, 173,819 images	728 patients, 1287 images	252 patients, 252 images	Sex	Binary	Sensitivity: 83.9% Specificity: 72.2% Accuracy: 78.6%
Mendoza et al., 2021 [27]	OCT	DL	1772 patients, 52,552 circle B-scans; 730 patients, 111,456 radial B-scans; 85% for training	5% for validation, 10% for testing	N/A	Age Axial length Sex Race Diabetes Hypertension CVD	Regression Regression Binary Binary Binary Binary Binary	MAE: 5.4, *R*^2^: 0.73 MAE: 0.7, *R*^2^: 0.3 AUC: 0.72 AUC: 0.96 AUC: 0.65 AUC: 0.71 AUC: 0.56
Munk et al., 2021 [28]	Fundus images, OCT	ResNet-152	16,196 participants, 135,667 fundus images; 5578 participants, 85,536 OCT scans; 80% for training	10% for validation, 10% for testing	N/A	Age Sex	Binary Regression	1. Fundus images: MAE: 6.328, AUC: 0.80 2. OCT cross sections: MAE: 5.625, AUC: 0.84 3. OCT volumes: MAE: 4.541, AUC: 0.90
Nusinovici et al., 2022 [29]	Retinal images	RetiAGE	36,432 participants, 116,312 images	Validation: 4048 participants, 12,924 images; Test: 10,171 participants, 32,318 images	UK Biobank: 56,301 participants	Age	Binary	AUC: 0.756
Poplin et al., 2018 [30]	Retinal images	Inception-v3	UK Biobank: 48,101 patients, 96,082 images; EyePACS: 236,234 patients, 1,682,938 images	UK Biobank: 12,026 patients, 24,008 images; EyePACS-2K: 999 patients, 1958 images	N/A	Age Gender Smoking status HbA1c BMI SBP DBP	Regression Binary Binary Regression Regression Regression Regression	MAE: 3.26, *R*^2^: 0.74 AUC: 0.97 AUC: 0.71 MAE: 1.39, *R*^2^: 0.09 MAE: 3.29, *R*^2^: 0.13 MAE: 11.35, *R*^2^: 0.36 MAE: 6.42, *R*^2^: 0.32
Rim et al., 2020 [31]	Retinal images	VGG-16	27,516 participants, 86,994 images	6879 participants, 21,698 images	Set 1: 4343 participants, 9324 images; Set 2: BES: 1060 participants, 4234 images; Set 3: SEED: 7726 participants, 63,275 images; Set 4: UK Biobank: 25,366 participants, 50,732 images	Sex Age BMM Height Bodyweight PBF BMI Creatinine DBP SBP Hematocrit Hemoglobin RBC count	Binary Regression Regression Regression Regression Regression Regression Regression Regression Regression Regression Regression Regression	AUC: 0.91, Accuracy: 0.85 MAE: 3.78, *R*^2^: 0.36 MAE: N/A, *R*^2^: N/A MAE: 5.48, *R*^2^: 0.23 MAE: 8.28, *R*^2^: 0.17 MAE: N/A, *R*^2^: N/A MAE: 2.90, *R*^2^: 0.06 MAE: 0.11, *R*^2^: 0.12 MAE: 8.09, *R*^2^: 0.2 3MAE: 13.20, *R*^2^: 0.19 MAE: N/A, *R*^2^: N/A MAE: N/A, *R*^2^: N/A MAE: N/A, *R*^2^: N/A
Tham et al., 2019 [32]	Fundus images	ResNet, DenseNet	13,937 participants, 25,637 images	3485 participants, 6830 images	N/A	HbA1c	Regression	MAE: 0.87%
Vaghefi et al., 2019 [33]	Retinal images	CNN	81,711 participants, 165,104 images; 60% for training	20% for validation, 20% for testing	N/A	Smoking	Binary	Accuracy: 88.88% Specificity: 93.87% Sensitivity: 62.62% AUC: 0.86
Yang et al., 2020 [34]	Retinal images	VGG-16	SEED: 9748 participants, 110,099 images; 80% for training	20% for testing	N/A	Race	Ternary	Accuracy: 95.1%
Zhang et al., 2020 [35]	Retinal images	Inception-v3	625 participants, 1222 images; 80% for training	10% for validation, 10% for testing	N/A	Hyperglycemia Hypertension Dyslipidemia Age Gender Drinking status Salty taste Smoking status BMI WHR HCT MCHC T-BIL D-BIL	Binary Binary Binary Binary Binary Binary Binary Binary Binary Binary Binary Binary Binary Binary	AUC: 0.880 AUC: 0.766 AUC: 0.703 AUC: 0.850 AUC: 0.704 AUC: 0.948 AUC: 0.809 AUC: 0.794 AUC: 0.731 AUC: 0.704 AUC: 0.759 AUC: 0.686 AUC: 0.764 AUC: 0.703
Al-Absi et al., 2022 [36]	Retinal images	ResNet-34	Qatar Biobank: 233 patients, 874 images; 250 HC, 931 images	5-fold cross-validation	N/A	CVD	Binary	Accuracy: 75.6%
Mellor et al., 2019 [37]	Fundus images	ResNet	4782 participants	5-fold cross-validation	N/A	CVD	Binary	AUC: 0.77
Chang et al., 2019 [38]	Retinal images	NASNet-Large	33,025 participants, 96,968 images	6597 participants, 13,373 images	N/A	FAD	Regression	MAE: 2.74
Ng et al., 2022 [39]	Retinal images	DLPPC	58 patients, 116 images; 80% for training	20% for testing	N/A	SpO_2_ LICUS PC OT CBT ABT	Binary Binary Binary Binary Binary Binary	AUC: 0.712 AUC: 0.731 AUC: 0.722 AUC: 0.581 AUC: 0.800 AUC: 0.767
Mueller et al., 2022 [40]	Fundus images	MIL	97 patients, 34 HC; 83,126 images for training	9237 images for validation	N/A	PAD	Binary	Accuracy: 0.837 *F*_1_-score: 0.883 AUC: 0.89
Chang et al., 2020 [41]	Retinal images	DL-FAS	5296 participants, 12,362 images	Validation: 647 participants, 1526 images; Test: 654 participants, 1520 images	N/A	Atherosclerosis	Binary	AUC: 0.713 Accuracy: 0.583 Sensitivity: 0.891 Specificity: 0.404
Barriada et al., 2022 [42]	Retinal images	VGG-16	76 patients, 152 images	5-fold cross-validation	N/A	CACS	Binary	Accuracy: 0.72 *F*_1_-score: 0.62
Rim et al., 2021 [43]	Retinal images	RetiCAC	15,911 participants, 36,034 images	3965 participants, 8930 images	Set 1: 8707 participants, 18,920 images; Set 2: 527 participants, 1054 images	CACS	Binary	AUC: 0.742
Son et al., 2020 [44]	Retinal images	Inception-v3	20,130 participants, 44,184 images; 80% for training	20% for training; 5-fold cross-validation	N/A	CACS	Binary	AUC: 83.2%
Dai et al., 2020 [45]	Retinal images	CNN	735 patients, 684 HC; 60% for training	20% for validation, 20% for testing; 5-fold cross-validation	N/A	Hypertension	Binary	Accuracy: 60.94% Specificity: 63.80% AUC: 0.6506
Lo et al., 2021 [46]	Fundus images	AML-Net	200 patient images, 200 HC; 70% for training	30% for validation	N/A	Mild hypertension	Binary	Accuracy: 93.75%
Islam et al., 2021 [47]	Retinal images	DiaNet	EyePACS: over 80,000 images; Qatar Biobank: 246 patients, 246 controls, total 1852 images	5-fold cross-validation	N/A	Diabetes	Binary	Accuracy: 84.47% Specificity: 83.06% AUC: 84.46%
Wang et al., 2022 [48]	Retinal images	CNN	10,766 images	N/M	N/A	Short-term readmission risk in diabetes	Binary	Specificity: 0.79 Accuracy: 0.837
Zhang et al., 2018 [49]	Fundus images	ResNet	79 patients, 79 HC; 80% for training	20% for testing	N/A	Diabetes	Binary	Accuracy: 84.7%
Abbasi-Sureshjani et al., 2018 [50]	Retinal images	ResNet	5791 HC images, 3133 T2DM; 80% for training	20% for validation	N/A	T2DM	Binary	*F*_1_-score: 0.758
Heslinga et al., 2020 [51]	Retinal images	VGG-19	1376 participants, 5222 images	Validation: 464 participants, 1802 images; Test: 496 participants, 1900 images;	N/A	T2DM	Binary	AUC: 0.746
Yun et al., 2022 [52]	Retinal images	ResNet-18	UK Biobank: 37,904 patients, 69,639 images	Test: 12,173 patients, 22,342 images; Validation: 12,185 patients, 22,394 images	6575 images	T2DM	Binary	AUC: 0.731 Sensitivity: 0.662 Specificity: 0.662
Cervera et al., 2021 [53]	Retinal images	Squeezenet v1.0	1081 patients, 17,028 images	121 patients, 1892 images; 5-fold cross-validation	N/A	DPN	Binary	AUC: 0.8013
Mitani et al., 2020 [54]	Retinal images	Inception-v4	UK Biobank: 40,041 participants, 80,006 images	Validation: 11,388 participants, 22,742 images; Test: 5734 participants, 11,457 images	N/A	Hemoglobin Anemia	Regression Binary	MAE: 0.67 AUC: 0.87
Wei et al., 2021 [55]	OCT	AneNet	17 patients, 221 images; 13 HC, 207 images	5-fold cross-validation	N/A	Anemia	Binary	Accuracy: 0.9865 Sensitivity: 0.9838 Specificity: 0.9594 AUC: 0.9983
Zhao et al., 2022 [56]	UWF Fundus images	ASModel_ UWF, ASModel_ CroppedUWF	2445 participants, 9221 images	Validation: 213 participants, 577 images; Test: 565 participants, 1730 images	N/A	Hemoglobin Anemia	Regression Binary	MAE: 0.83 AUC: 0.93 Sensitivity: 91.2% Specificity: 80.00%
Kang et al., 2020 [57]	Retinal images	VGG-19	4970 patients, 20,787 images	Validation: 621 patients, 2189 images; Test: 621 patients, 2730 images	N/A	Early renal function impairment	Binary	AUC: 0.81 Sensitivity: 0.83 Specificity: 0.62 Accuracy: 0.73
Sabanayagam et al., 2020 [58]	Retinal images	CondenseNet	SEED: 5188 participants, 10,376 images	1297 participants, 2594 images; 5-fold cross-validation	1. 3735 participants, 7470 images; 2. BES: 1538 participants, 3076 images	CKD	Binary	AUC: 0.835 Sensitivity: 0.75 Specificity: 0.75
Zhang et al., 2021 [59]	Retinal images	ResNet-50	30,122 participants, 60,244 images	Validation: 4307 participants, 8614 images; Test: 8727 participants, 17,454 images	1. 8059 participants, 16,118 images; 2. 3081 participants, 6162 images	CKD Early CKD T2DM	Binary Binary Binary	AUC: 0.885 AUC: 0.834 AUC: 0.854
Xiao et al., 2021 [19]	Retinal imagesSlit-lamp images	ResNet-101	1252 participants, 2481 slit-lamp images, 1989 retinal images; 75% for training	25% for tuning	537 participants, 1069 slit-lamp images, 800 retinal images	Liver cancer Liver cirrhosis Chronic viral hepatitis Non-alcoholic fatty liver disease Cholelithiasis Hepatic cyst	Binary Binary Binary Binary Binary Binary	Slit-lamp; Retinal images: AUC: 0.93; 0.84 AUC: 0.90; 0.83 AUC: 0.69; 0.62 AUC: 0.63; 0.70 AUC: 0.58; 0.68 AUC: 0.66; 0.69
Cho et al., 2022 [60]	Retinal images	DenseNet-201, EfficientNet-B7	1703 patients, 3353 images	189 patients, 373 images; 10-fold cross-validation	N/A	WMH	Binary	Sensitivity: 66.1 Specificity: 71.3 AUC: 0.736
Appaji et al., 2022 [61]	Retinal images	CNN	116 patients, 82 HC	Validation: 33 patients, 23 HC; Test: 17 patients, 13 HC; Confirmatory: 21 patients, 22 HC	N/A	SCZ	Binary	Accuracy: 95% AUC: 0.98Sensitivity: 91.66% Specificity: 95%
Lai et al., 2020 [62]	Retinal images	ResNet-50	46 patients, 24 HC	10-fold cross-validation	N/A	ASD	Binary	Sensitivity: 82.6% Specificity: 91.3% AUC: 0.907
Wisely et al., 2019 [63]	Retinal images	ResNet-18	36 patients, 117 HC for all datasets; 57 patient eyes, 198 HC eyes for training	6 patient eyes, 24 control eyes for testing; 9-fold cross validation	N/A	AD	Binary	AUC: 0.74 Accuracy: 0.79
Huang et al., 2020 [64]	Retinal images	EfficientNet-B1	144 patients, 74 HC, total 342 images; Training and validation: 187 participants	Training and validation: 187 participants Testing: 31 participants	N/A	Axial spondyloarthritis	Binary	AUC: 0.735 Sensitivity: 87% Specificity: 62.5%

^1^ Only the best performance is presented when there was more than one model. Metadata-based models and hybrid models are not presented in this table. SEED, Singapore Epidemiology of Eye Diseases; N/A, not applicable; AUC, area under curve; SBP, systolic blood pressure; DBP, diastolic blood pressure; BMI, body mass index; APOE4, apolipoprotein E4; MAE, mean absolute error; RFM, relative fat mass; SHBG, sex hormone binding globulin; Tch, total cholesterol; HDL, high density lipoprotein; LDL, low density lipoprotein; Tg, triglyceride; ARB, angiotensin receptor blocker; ACEi, angiotensin-converting enzyme inhibitor; HC, healthy control; DM, diabetes mellitus; OCT, optical coherence tomography; CVD, cardiovascular diseases; EyePACS, eye picture archive communication system; BES, Beijing Eye Study; BMM, body muscle mass; PBF, percentage body fat; RBC, red blood cell; WHR, waist–hip ratio; HCT, hematocrit; MCHC, mean corpuscular hemoglobin concentration; T-BIL, total bilirubin; D-BIL, direct bilirubin; FAD, fundus age difference; SpO_2_, oxygen saturation; LICUS, length of ICU stay; PC, perioperative complications; OT, operation time; CBT, cardiopulmonary bypass time; ABT, arterial blocking time; PAD, peripheral arterial disease; CACS, coronary artery calcium score; N/M, not mentioned; T2DM, type 2 diabetes mellitus; DPN, diabetic peripheral neuropathy; UWF, ultra-wide-field; CKD, chronic kidney disease; WMH, white matter hyperintensity; SCZ, schizophrenia; ASD, autistic spectrum disorder; AD, Alzheimer disease.

#### 3.3.1. Systemic Health Features

Systemic health features, such as age, gender, smoking status, blood pressure, and glucose level, are indicative and predictive of various disorders. The pioneering work of Poplin et al. [30] unveiled the possibility of using deep learning algorithms based on fundus photographs to predict systemic risk factors, giving rise to a series of works with akin goals. These models successfully predicted age with the mean absolute error (MAE) ranging from 2.43 to 6.328 [22,24,25,28,30,31]. As for identifying gender, the models also achieved satisfying AUCs ranging from 0.85 to 0.97 [20,22,24,25,26,28,30,31,52], with a few studies highlighting the optic disc and the macula as regions of interest [20,26,30]. Ethnicities could also be categorized from fundus images with AUC and accuracy surpassing 0.90 [24,34].

Other than fundus images, OCT scans were also proven to be suggestive of patients’ age and gender. The result from Munk et al. [28] indicated that prediction performance with OCT C-Scans or B-Scans of the macular region outperformed fundus images, achieving AUCs of 0.90 and 0.84 in detecting gender and obtaining the MAEs of 5.625 and 4.541 in predicting age. Mendoza et al. [27] proposed that circle and radial scans of the optic nerve head incorporate the potential for predicting age, gender, and race, among which race prediction achieved the best performance with an AUC of 0.96. The authors also illustrated that circle scans have better predictive value in DL algorithms.

As a notorious risk factor for various systemic diseases, smoking is deleterious since it impacts systemic vascular structure and function [65]. Recent studies have demonstrated that DL diagnostic models based on retinal images could achieve AUCs between 0.71 and 0.78 by capturing the pathological changes, as retinal circulation characteristics were marked in the attention maps [22,24,30]. Contrast-enhanced photographs emphasizing the vessel structure could significantly boost the model’s performance and reach an accuracy of 88.88% [33].

Based on the parallel decrepitude of the body and the retina, several researchers proposed the idea of “retinal age” as a novel feature in disease monitoring. Chang et al. [38] suggested that a higher algorithm-predicted age than the chronological age translates into higher all-cause mortality. Nusinovici et al. [29] interpreted it with a different approach by defining “RetiAGE” as the probability of age being ≥65 years, and their study obtained similar results. The work of Hu et al. [23] extended the application of this method, proving that the model based on retinal age is predictive of the future risk of Parkinson’s disease with an AUC of 0.71.

#### 3.3.2. CVD

Cardiovascular diseases (CVD) cause the most significant proportion of deaths among non-communicable diseases. Studies have proven that the presence and severity of CVD are associated with retinal vascular morphology [66], providing the theoretical basis for building AI diagnosing models with retinal images.

The coronary artery calcium score (CACS) is a non-invasive assessment system that quantifies the prognostic risks of CVD [67]. Two studies have applied DL to predict CAC cores based on retinal images. One of the algorithms attained the highest accuracy of 0.72 in predicting CACS >0 despite a small sample size [42], and both studies suggested that the AUC improved as the diagnosing threshold increased [44].

Alternatively, several researchers have developed unique retinal scoring systems to use as CVD indicators. The RetiCAC score, the probability score of the DL binary classification task, could predict the presence of coronary artery calcium with an AUC over 0.70 [43] and was comparable with the traditional CAC risk stratification in predicting disease prognosis. Another scoring system based on retinal images, namely the DL fundoscopic atherosclerosis score (DL-FAS), achieved akin results in predicting carotid artery atherosclerosis and all-cause mortality [41].

For direct classification of CVD, Al-Absi et al. [36] achieved an accuracy of 75.6% using only retinal images, and the region of interest of the model was mainly the central retinal area. Another study recruiting type 1 diabetes mellitus patients achieved an AUC of 0.77 in diagnosing CVD [37]. Peripheral artery disease (PAD), also attributed to atherosclerosis, was proven to be detectable from fundus images with an AUC reaching 0.89 [40]. Furthermore, there was evidence of applying retinal image-based AI in predicting perioperative parameters of congenital heart diseases [39], with the AUC of detecting cardiopulmonary bypass time reaching 0.80 and that of oxygen saturation, arterial blocking time, length of ICU stay, and perioperative complications surpassing 0.70.

#### 3.3.3. Hypertension

Hypertension causes microvascular dysfunction. Morphological retinal vascular changes, such as narrower arteries and wider venules, could be observed in hypertensive patients [68]. In algorithms predicting biomarkers, the MAE was from 8.96 to 11.35 for systolic blood pressure (BP) and 6.42 to 7.20 for diastolic BP [22,30,31]. Interestingly, the studies applying DL to diagnose hypertension concomitantly preprocessed the input photographs to augment the vessel structures and erase background noise. The models based on processed images achieved AUC values of 0.65% and 0.77%, respectively [35,45], and the work predicting mild hypertension reached an accuracy of 93.75% [46] based on only 400 photographs.

#### 3.3.4. Diabetes Mellitus

Diabetic retinopathy, with its rocketing prevalence and distinct fundus pathologies, has become the pilot field of ophthalmic AI. Aside from diagnosing typical retinopathy, there have been multiple attempts at employing DL to predict diabetic mellitus (DM) as a disease. Kang et al. reached the highest AUC of 0.92 [59], and the performance of other approaches was no worse than 0.73 [35,47,50,51,52]. When evaluated for accuracy, the models reached from 83.7% to 85.0% [48,49]. One study that applied Xception and dense neural network (DNN) achieved a training accuracy of 96.68% and a validation accuracy of 66.82% although only 220 images were put into model training.

Hemoglobin A1c (HbA1c) is an essential biomarker for long-term glucose monitoring [69]. Tham et al. have proven that retinal images contain information indicating HbA1c level by achieving an MAE of 0.87% with the DL algorithm [32]. Notably, it was suggested that diabetic neuropathy could also be detected from fundus photographs, with the AUC reaching 0.71 [53].

#### 3.3.5. Anemia

Anemia is a common disease and a symptom of various systemic disorders. DL based on fundus images was proven sufficient in predicting hemoglobin concentration and diagnosing anemia [54,56], thus could be considered a novel non-invasive method for disease management. Explanation methods showed that the models focused on the optic disc and the retinal vessels, which is consistent with the typical ocular symptoms such as pale discs and narrower arteries in anemic patients.

Wei et al. [55] tackled the problem from a different perspective by using OCT images that displayed the cross-section of retinal vessels as the model input. Although the algorithm achieved excellent results, the dataset was diminutive and external validation was not applied.

#### 3.3.6. Hepatobiliary Diseases and Kidney Diseases

The liver and the kidney share multiple essential physiological functions, including metabolism and maintaining homeostasis. Recent studies have suggested that diseases of both organs can be observed with deep learning algorithms based on fundus photos. Xiao et al. [19] proved that hepatobiliary diseases, especially liver cancer and liver cirrhosis, could be diagnosed with an AUC over 0.80 from fundus images. In the case of chronic kidney disease (CKD), the algorithms obtained excellent performance in predicting early CKD and CKD [57,58,59]. Color fundus images could provide intuitive observation of the systemic microvasculature, enabling the detection of vascular defects in CKD patients.

#### 3.3.7. Neurological Disorders

A diversity of neurological diseases can be detected from the morphological changes of the retina. White matter hyperintensity, referring to the lesions caused by cerebral small vessel diseases, is predicted from fundus photos with an AUC of approximately 0.70 [60]. As for cognitive impairment, previous studies indicated that DL with retinal images alone was limited in predicting cognition status [21]; however, UWF combined with OCTA and autofluorescence (FAF) could achieve an AUC of 0.74 in detecting Alzheimer’s disease (AD) [63]. Likewise, autoimmune diseases such as axial spondyloarthritis [64] could also be distinguished with a fair AUC of 0.74. On the contrary, studies focusing on autism spectrum disorder (ASD) [62] and schizophrenia [61] obtained an AUC of over 0.97, possibly attributable to the fact that both models applied cross-validation methods for performance evaluation. These results have proven that several categories of neurological disorders demonstrate retinal changes, although the DL models based on fundus images are not yet sufficiently developed to perform diagnostic tasks individually.

### 3.4. Algorithms Based on the Movements of the Eye

Eye movements are coordinative actions dominated by cognitive processes and behavior mechanisms [70]. Previous studies have proven that the specific gaze patterns captured by eye-tracking devices could be predictive of neurodegenerative diseases, such as Parkinson’s disease (PD), dementia, and autism spectrum disorders (ASD). With the advancements in hardware and algorithms, the current eye-tracking methods have achieved explicit temporal resolutions and could provide additional information unattainable by traditional imaging techniques.

#### 3.4.1. Dementia and Parkinson’s Disease

Dementia is a global health issue in the aging society. It was suggested that eye-tracking tests could provide a rapid and objective method for assessing patients’ cognitive functions, such as memory and attention [11]. Mengoudi et al. [71] designed a test to trace the participants’ sight while presenting images with different stimuli, and the model achieved an accuracy of 78.3% in classifying dementia. Alternatively, Biondi et al. [72] developed a resolution based on eye movement during reading tasks. Their result had a decent performance with an accuracy of 89.8%, and the severity scaled by model output was comparable with psychiatrists’ scoring.

PD is another neurodegenerative disease affecting a large population worldwide. As previous studies implied fixational defects in PD patients, Archila et al. [73] developed an algorithm based on fixational performances to distinguish and stage PD. Their model achieved relatively good specificities, and the performance advanced after combining gait data.

**Table 3:** Summary of deep learning algorithms identifying systemic diseases from eye movements.

Author, Year	Ocular Data	DL Model	Training Dataset	Testing/Validation Dataset	External Validation	Systemic Health Features/Diseases	Outcome	Performance ^1^
Li et al., 2022 [74]	Gaze estimation videos	AttentionGazeNet, LSTM	50 participants, 64,000 images	1. 15 participants, about 1500 images; 2. 16 participants	405 participants, 405 videos	ASD	Binary	Accuracy: 94.8% Sensitivity: 91.1% Specificity: 96.7%
Li et al., 2020 [75]	Eye movement videos	LSTM	136 patients, 136 videos; 136 HC, 136 videos	10-fold cross-validation	N/A	ASD	Binary	Accuracy: 92.7% Sensitivity: 91.9% Specificity: 93.4%
Varma et al., 2022 [76]	Eye movement videos	LSTM	68 patients and 27 HC in all datasets; 324 videos for training	Validation: 71 videos; Test: 54 videos	N/A	ASD	Binary	Recall: 0.656 Precision: 0.661
Xie et al., 2022 [77]	Eye movement data	VGG-16	20 patients, 19 HC	Leave-one-out and 13-fold cross-validation	N/A	ASD	Binary	Accuracy: 0.95 Sensitivity: 1.00 Specificity: 0.89 AUC: 0.93
Jiang et al., 2017 [78]	Eye movement data	VGG-16	39 participants, 100 images	Leave-one-subject-out cross-validation	N/A	ASD	Binary	Accuracy: 0.92 Sensitivity: 0.93 Specificity: 0.92 AUC: 0.92
Mengoudi et al., 2020 [71]	Eye movement data	Self-Supervised Learning, SVM	432 HC	30 patients, 144 HC	N/A	Dementia	Binary	Accuracy: 78.3% Sensitivity: 89.7% Specificity: 67.6%
Biondi et al., 2018 [72]	Eye movement data	Sparse-Autoencoders	22 patients, 39 HC, total 2922 trials	4 patients, 4 HC, total 313 trials	N/A	AD	Binary	Accuracy: 89.78%
Archila et al., 2021 [73]	Eye movement videos	LSTM	12 patients, 144 videos; 13 HC, 156 videos	Leave-one-patient-out cross-validation	N/A	PD	Ternary	Specificity: Control: 1, Stage2: 0.87, Stage3: 0.86 *F*_1_-score: Control: 0.81, Stage2: 0.57, Stage3: 0.72
Mao et al., 2020 [79]	Eye movement data	LSTM	34 HC, 34 patients with brain injury, and 30 patients with vertigo; 64 subjects for training	34 subjects for testing	N/A	Brain injury and vertigo	Ternary	Accuracy: 0.9412
Ahmadi et al., 2020 [80]	Eye movement data	RF, ANN, SingleGMC, MultiGMC	40 patients with vestibular stroke, 68 patients with peripheral AVS; 90% for training	10% for testing	N/A	AVS	Binary	Accuracy: 82% AUC: 0.96

^1^ Only the best performance is presented when there was more than one model. Metadata-based models and hybrid models are not presented in this table. ASD, autism spectrum disorder; HC, healthy control; N/A, not applicable; AUC, area under curve; AD, Alzheimer disease; PD, Parkinson’s disease; AVS, acute vestibular syndrome.

#### 3.4.2. Autism Spectrum Disorders

Visual attention characteristics are among the most specific traits obtained from eye movement data. Such hallmarks could be applied in ASD screening, which distinctively presents changes in attention patterns towards certain visual elements. Jiang et al. [78] discovered that ASD patients mainly focused on non-social subjects while presented with a variety of pictures, and their model achieved an AUC of 0.92. Xie et al. [77] further distinguished several categories of image features, such as outdoor objects and food and drinks, that poses importance in identifying ASD. The model based on the top features also performed excellently with an AUC of 0.92.

Li et al. adopted a different method by displaying the mother’s image and tracking the children’s gaze patterns [74,75]. By applying appearance-based gaze estimation, their models achieved high accuracies of over 90%. Besides the reaction to still images, Varma et al. [76] captured the gaze fixation and visual scanning methods in socially motivated gameplay. The developed algorithm showed mild predictive power in identifying ASD in children.

#### 3.4.3. Other Disorders

Vestibular disorders could cause significant ocular presentations, namely abnormal nystagmus and saccade. It usually requires an experienced specialist for evaluation in clinical settings to help diagnose vestibular diseases. With the advancement of DL, a few studies utilized eye movement data for discrimination between systemic diseases. Ahmadi et al. [80] identified vestibular strokes and peripheral acute vestibular syndrome with an AUC of 0.96. Mao et al. [79], on the other hand, obtained eye motion during gazing tasks and achieved an AUC of 0.94 in differentiating controls, brain injury, and vertigo patients.

## 4. Discussion

This systematic review concludes the performances of deep learning algorithms based on ocular data in evaluating systemic health conditions. Overall, most systemic diseases proven to be detectable from static ocular manifestations impact neurovascular structures, which project changes to the eye in areas such as retinal vessels and corneal nerves. Most studies used colored photographs as input; however, depth-resoluted OCT images were also applicable. Alternatively, neurodegenerative disorders mainly present as defects in eye movements, and eye-tracking data in specific tasks or spontaneous abnormalities were obtained as model inputs. The reported algorithms achieved fair results, with AUCs and accuracies exceeding 0.7 in most studies despite small datasets. The saliency maps and heatmaps also showed that the models were built on rational reasoning despite the “black box” process of deep learning. Regardless of the outstanding performances presented in mostly retrospective datasets and with handpicked participants, several aspects should be advanced before putting the models in real-world application.

### 4.1. Present and Prospects

Systemic health features could have significant latent effects on the primeval ocular characteristics. Features such as age, sex, and ethnicity were proven to be credibly identified from ophthalmic data. While predicting age, the algorithms mainly focused on retinal vessels and the optic nerve head (ONH) areas [25,30], which are concordant with the aging of the retina [81,82]. Sex, on the other hand, was identified based on the ONH and the macular area, where innate gender differences in ONH blood supply [83] and FAZ area [84] exist. Besides being the baseline characteristics of the patients, these features could concurrently be risk factors for many systemic diseases. Former reports [7,8] proved that age and sex are interrelated with cardiometabolic risk factors and conditions in retinal image-based DL algorithms, possibly due to their mutual effect on fundus vessels. Therefore, studies targeting diseases with sex or age differences should control for these confounders to prevent overestimating the model’s performance.

Ethnicity was another critical factor proven to be distinguishable from ophthalmic presentations. Aside from affecting the retinal structure [85], ethnicity is also a determinant of the ocular disease spectrum. However, most algorithms were trained on datasets with little to no diversity, affecting the generalizability in real-world scenarios. We suggest that researchers consider data with racial diversity as external validation, and more multi-ethnic datasets should be established to produce generalizable DL models.

Regarding algorithms for diagnosing neurodegenerative diseases based on gazing patterns and eye movements, the communal issues are the limited datasets and the lack of external validation. With video data as input in most cases, these algorithms must be robust against significantly greater interferences to be applied in different real-world scenarios. A large-scale validation in the generalized public would be much preferred for further approval of the DL algorithms.

DL is known for its representation-learning nature. The ocular vasculature, including the conjunctival and the retinal vessels, were some of the most conspicuous and vulnerable structures and were often identified as the focused feature in saliency maps. For instance, metabolic syndrome [86] presented as hypertension, hyperglycemia, and dyslipidemia was found to cause retinal arteriolar narrowing [87]. Arterial defects in these conditions were reported to be caused by a few shared pathophysiology, such as oxidative stress, glutathione peroxidase, and impaired acetylcholine-mediated vasodilatation. As a result, algorithms predicting hypertension, diabetes, and CVD simultaneously highlighted the retinal vessels as the area of interest. On the other hand, CKD causes systemic atherosclerosis and vascular calcification [88], which could also present in the retina as arteriolar thinning and sparse capillaries. Since current studies mainly focused on discriminating the target disease from healthy controls, the algorithms were likely to identify universal pathologies instead of exclusive characteristics of each condition. Therefore, these DL models could lack specificity if applied in real-world scenarios where all systemic diseases coexist. Future studies aiming to distinguish between diseases with similar pathological characteristics would greatly favor the implementation of DL algorithms in real-world screening and diagnosis.

### 4.2. Advantages and Drawbacks of AI in Clinical Settings

The application of AI algorithms in clinical settings has been a controversial topic. AI models benefit disease screening, diagnosing, and management in several aspects: (a) improve efficiency compared with human graders and enable large-scale screening programs; (b) allow advanced medical technology to reach remote areas with algorithms deployed in portable devices; (c) reduce health-care expenses by saving human resources; and (d) discover preclinical changes for early disease screening. Implementing ophthalmic examinations in disease screening algorithms further provides several advantages. Ophthalmic examinations are non-invasive and rapid compared with other traditional tests; therefore, the screening procedure can be simplified to a great extent. Moreover, the neurovascular structures could be observed intuitively from the ocular anatomy, offering a window for analyzing the underlying morphological and pathological features.

Nonetheless, there are primary disputes about launching AI algorithms in clinical settings. First and foremost is the debate on AI ethics. Models should be thoroughly investigated before being assigned with allowance for real-world tasks. Secondly, the robustness of AI models is often questioned in actual practice. Despite data with varied baseline characteristics, the algorithms could also encounter a variety of low-quality inputs. Researchers should ensure the algorithm can adapt to widely-varied datasets to offer a generalizable and reliable program.

### 4.3. Strengths and Limitations

This systematic review is the first to conclude deep learning algorithms for systemic disease screening and diagnosing based on ocular data. It provides a comprehensive view of the current trend and methodology in observing various systemic conditions from eye manifestations. We believe this work could be a valuable reference for subsequent studies.

There are some limitations in the current study. According to our selection criteria, studies utilizing DL for feature extraction and statistical methods for condition diagnosis were excluded. This may lead to information loss, as several studies achieving decent results were eliminated. Secondly, our study included meeting abstracts to involve up-to-date research works that have not yet been published. However, the lack of detailed information in the study design translates into unknown risks of bias. Lastly, this review did not inspect the development of deep learning algorithms in detail. Future reviews focusing on AI techniques are preferred to provide further information for computer scientists and program developers.

## 5. Conclusions

Deep learning has been shown to be beneficial in identifying systemic diseases from ocular presentations. Despite presenting decent performance in the articles, the algorithms still have several shortcomings for clinical application. Future studies should aim at improving the disease specificity and generalizability of the DL models for implementation in real-world screening tasks.

## Data Availability

Not applicable.

## Supplementary Materials

The following supporting information can be downloaded at: https://www.mdpi.com/article/10.3390/diagnostics13050900/s1; Table S1: The search strategy used for obtaining research articles in the three selected databases; Table S2: The detailed results of the QUADAS-2 analysis.
