# Supplementary material for: Deep Learning Algorithms for Screening and Diagnosis of Systemic Diseases Based on Ophthalmic Manifestations: A Systematic Review
Source: Diagnostics (Basel). 2023 Feb 27;13(5):900. doi: 10.3390/diagnostics13050900 (PMC10001234; doi:10.3390/diagnostics13050900)
Supplement: Supplementary file 1 [file diagnostics-13-00900-s001.zip › Supplementary Table S1.pdf]

Supplementary Table S1. The search strategy used for obtaining research articles in the three selected databases.

Database	Categories	Keywords
PubMed	Ocular Characteristics	"eye"[MeSH Terms] OR "eye"[Title/Abstract] OR "eyes"[Title/Abstract] OR "microscopy, confocal"[MeSH Terms] OR (("fundus oculi"[MeSH Terms] OR "retina"[MeSH Terms] OR "retinal"[Title/Abstract] OR "fundus"[Title/Abstract]) AND ("photography"[MeSH Terms] OR "photography"[Title/Abstract] OR "photographies"[Title/Abstract] OR "photo"[Title/Abstract] OR "photos"[Title/Abstract] OR "photograph"[Title/Abstract] OR "photographs"[Title/Abstract] OR "image"[Title/Abstract] OR "images"[Title/Abstract] OR "imaging"[Title/Abstract])) OR "tomography, optical coherence"[MeSH Terms] OR "optical coherence tomography"[Title/Abstract] OR "oct"[Title/Abstract] OR "slit lamp microscopy"[MeSH Terms]
	Systemic Diseases	"diseases category"[MeSH Terms]
	Deep Learning	"deep learning"[MeSH Terms] OR "deep learning"[Title/Abstract])) NOT ("review"[Publication Type] OR "systematic review"[Publication Type] OR "editorial"[Publication Type] OR "letter"[Publication Type] OR "biography"[Publication Type] OR "retracted publication"[Publication Type] OR "meta analysis"[Publication Type] OR "case reports"[Publication Type]
	Other	"humans"[MeSH Terms]
Embase	Ocular Characteristics	'eye'/exp/mj OR 'eyes'/exp/mj OR 'visual system function'/exp/mj OR 'ophthalmology'/exp/mj OR 'eye examination'/exp OR 'visual system examination'/exp OR 'ophthalmological diagnostic device'/exp OR 'confocal microscopy'/exp OR 'optical coherence tomography'/exp OR 'oct'/exp OR 'retinal imaging'/exp OR 'retinal photography'/exp OR 'retinal camera'/exp OR 'fundus photography'/exp OR 'fundus camera'/exp OR 'slit lamp microscopy'/exp)
	Systemic Diseases	'diseases'/exp/mj
	Deep Learning	'deep learning'/exp/mj OR 'deep learning':ab,ti
	Other	[humans]/lim NOT ([systematic review]/lim OR [meta analysis]/lim OR [editorial]/lim OR [letter]/lim OR [review]/lim OR 'biography':it OR 'retracted publication':it OR 'case report':it
Web of Science	Ocular Characteristics	TITLE-ABS-KEY (eye OR ocular OR fundus OR retinal OR corneal OR "retinal vessel" OR "confocal microscopy" OR "optical coherence tomography" OR "oct" OR "slit lamp")
	Systemic Diseases	TITLE-ABS-KEY (biomarkers OR "systemic diseases" OR cerebrovascular OR alzheimers OR dementia OR "white matter hyperintensities" OR neurodegenerative OR psychiatric OR thyroid OR cardiovascular OR pulmonary OR coronary OR heart OR anemia OR hypertension OR diabetes OR liver OR hepatobiliary OR "chornic kidney disease" OR pediatric OR gestational OR "covid 19")
	Deep Learning	TITLE-ABS-KEY ("deep learning" OR "deep-learning")
	Other	EXCLUDE (DOCTYPE , "re") OR EXCLUDE (DOCTYPE , "ed") OR EXCLUDE (DOCTYPE , "le") OR EXCLUDE (DOCTYPE , "tb")
