# Supplementary material for: Deep Learning Algorithms for Screening and Diagnosis of Systemic Diseases Based on Ophthalmic Manifestations: A Systematic Review
Source: Diagnostics (Basel). 2023 Feb 27;13(5):900. doi: 10.3390/diagnostics13050900 (PMC10001234; doi:10.3390/diagnostics13050900)
Supplement: Supplementary file 1 [file diagnostics-13-00900-s001.zip › Supplementary Table S2.pdf]

Table S2. The detailed result of the QUADAS-2 analysis.

Author, Publication Year	Risk of Bias				Applicability Concerns		
	Patient Selection	Index Test	Reference Standard	Flow and Timing	Patient Selection	Index Test	Reference Standard
Babenko et al, 2022	☺	☺	☺	☺	☺	☺	☺
Li et al, 2022	☹	☺	☺	☺	☹	☺	☺
Preston et al, 2022	☺	☺	☺	☺	☺	☺	☺
Scarpa et al, 2020	☹	☺	☺	☺	☺	☺	☺
Althnian et al, 2021	☹	☹	☺	☺	☹	☹	☺
Lv et al, 2021	☹	☺	☺	☺	☺	☺	☺
Betzler et al, 2021	☺	☺	☺	☺	☺	☺	☺
Corbin et al, 2022	☺	☺	☺	☺	☺	☺	☺
Gerrits et al, 2020	☺	☺	☺	☺	☺	☺	☺
Hu et al, 2022	☺	☹	☺	☺	☺	☹	☺
Khan et al, 2022	☺	☺	☺	☹	☺	☺	☺
Kim et al, 2020	☺	☹	☺	☺	☺	☹	☺
Korot et al, 2021	☺	☹	☺	☺	☺	☹	☺
Mendoza et al, 2021	?	?	☺	☺	?	?	☺
Munk et al, 2021	☺	☺	☺	☺	☺	☺	☺
Nusinovici et al, 2022	☺	☺	☺	☺	☺	☺	☺
Poplin et al, 2018	☺	☺	☺	☺	☺	☺	☺
Rim et al, 2020	☺	☺	☺	☺	☺	☺	☺
Tham et al, 2019	☺	?	?	?	☺	?	?
Vaghefi et al, 2019	☺	☺	☺	☺	☺	☺	☺
Yang et al, 2020	☺	☺	☺	☺	☺	☺	☺
Zhang et al, 2020	☺	☺	☹	☺	☺	☺	☹
Al-Absi et al, 2022	☺	☺	☺	☺	☺	☺	☺

Mellor et al, 2019	☺	?	?	?	☺	?	?
Chang et al, 2019	☺	☹	☺	☺	☺	☹	☺
Ng et al, 2022	☹	☹	☺	☺	☹	☹	☺
Mueller et al, 2022	☹	☺	☺	☺	☹	☺	☺
Chang et al, 2020	☺	☺	☺	☺	☺	☺	☺
Barriada et al, 2022	☹	☺	☺	☺	☹	☺	☺
Rim et al, 2021	☺	☺	☺	☺	☺	☺	☺
Son et al, 2020	☺	☺	☺	☺	☺	☹	☺
Dai et al, 2020	☺	☺	☺	☺	☺	☺	☺
Lo et al, 2021	☺	☹	☺	☺	☺	☹	☺
Islam et al, 2021	☺	☺	☺	☺	☺	☺	☺
Wang et al, 2022	☺	☹	☹	☹	☺	☹	☹
Zhang et al, 2018	☹	☹	?	?	☹	☹	☹
Abbasi-Sureshjani et al, 2018	☺	☹	☹	☺	☺	☹	☹
Heslinga et al, 2020	☹	☺	☺	☺	☹	☺	☺
Yun et al, 2022	☺	☺	☺	☺	☺	☺	☺
Cervera et al, 2021	☺	☺	☺	☺	☺	☺	☺
Mitani et al, 2020	☺	☺	☺	☺	☺	☺	☺
Wei et al, 2021	☺	☺	☺	☺	☺	☺	☺
Zhao et al, 2022	☺	☺	☺	☺	☺	☺	☺
Kang et al, 2020	☺	☺	☺	☺	☺	☺	☺
Sabanayagam et al, 2020	☺	☺	☺	☺	☺	☺	☺
Zhang et al, 2021	☺	☺	☺	☺	☺	☺	☺
Xiao et al, 2021	☺	☺	☺	☺	☺	☺	☺
Cho et al, 2022	☺	☺	☺	☹	☺	☺	☺
Appaji et al, 2022	☹	☹	☹	☺	☹	☹	☹
Lai et al, 2020	☹	☺	☺	☺	☹	☺	☺
Wisely et al, 2019	☹	☺	☹	☺	☹	☺	☹
Huang et al, 2020	?	?	☺	?	?	☺	?

Li et al, 2022	☺	☺	☺	☺	☺	☺	☺
Li et al, 2020	☹	☺	☺	☺	☺	☺	☺
Varma et al, 2022	☹	☺	☺	☺	☺	☺	☺
Xie et al, 2022	☺	☹	☺	☺	☺	☹	☺
Jiang et al, 2017	?	☺	☺	☺	?	☺	☺
Mengoudi et al, 2020	☹	☺	☺	☺	☹	☺	☺
Biondi et al, 2018	☺	☺	☺	☺	☺	☺	☺
Archila et al, 2021	☺	☹	☺	☺	☺	☹	☺
Mao et al, 2020	☺	☹	☺	☺	☺	☹	☺
Ahmadi et al, 2020	☺	☹	☺	☺	☺	☹	☺

☺ = low risk; ☹ = high risk; ? = unclear risk
